# Head and Neck Sarcomas: A Comprehensive Cancer Center Experience

**DOI:** 10.3390/cancers5030890

**Published:** 2013-07-15

**Authors:** Mohamedtaki A. Tejani, Thomas J. Galloway, Miriam Lango, John A. Ridge, Margaret von Mehren

**Affiliations:** 1 Division of Hematology/Oncology, University of Rochester Medical Center, Rochester, NY 14642, USA; E-Mail: mohamed_tejani@urmc.rochester.edu; 2 Department of Radiation Oncology, Fox Chase Cancer Center, Philadelphia, PA 19111, USA; E-Mail: thomas.galloway@fccc.edu; 3 Department of Surgical Oncology, Fox Chase Cancer Center, Philadelphia, PA 19111, USA; E-Mails: miriam.lango@fccc.edu (M.L.); drew.ridge@fccc.edu (J.A.R.); 4 Department of Medical Oncology, Fox Chase Cancer Center, Philadelphia, PA 19111, USA

**Keywords:** head and neck sarcomas, soft tissue sarcomas, osteogenic sarcomas

## Abstract

Head/neck sarcomas are rare, accounting for about 1% of head/neck malignancies and 5% of sarcomas. Outcomes have historically been worse in this group, due to anatomic constraints leading to difficulty in completely excising tumors, with high rates of local recurrence. We retrospectively analyzed cases of head/neck soft tissue sarcomas (STS) and osteogenic sarcomas managed in a multi-disciplinary setting at Fox Chase Cancer Center from 1999–2009 to describe clinicopathologic characteristics, treatment, outcomes, and prognostic factors for disease control and survival. Thirty patients with STS and seven patients with osteogenic sarcoma were identified. Most STS were high grade (23) and almost all were localized at presentation (28). Common histologies were synovial cell (6), rhabdomyosarcoma (5), angiosarcoma (4), liposarcoma (4) and leiomyosarcoma (3). The type of primary therapy and disease outcomes were analyzed. Cox proportional hazards regression analysis was performed to identify predictors of disease-free survival (DFS) and overall survival (OS). The HR and 95% CI for Cox model and median DFS/OS analyzed by Kaplan-Meier curves were calculated.

## 1. Introduction

Head and neck sarcomas are rare tumors, accounting for only 1% of all head and neck malignancies and 5% of all sarcomas [1,2]. They comprise about 1,000 to 1,500 cases per year in the United States and the many histologic subtypes make rigorous study of these neoplasms difficult. Diagnostic and treatment algorithms are mainly based on expert consensus and retrospective case series. The experiences depend strongly on the reporting entity—patients predominantly seen by head and neck surgeons, pediatric oncologists and medical oncologists will have disparate histologies and clinical features. Our study focuses solely on adults treated in multi-disciplinary fashion by surgical, radiation and medical oncologists at Fox Chase Cancer Center.

In general, the natural history of these sarcomas parallels that of their non-head and neck counterparts, but with a higher reported rate of local recurrence and lower overall survival rate. In a single institution series of 102 patients with head and neck sarcomas between 1960 and 1999 [3], local control rates were inferior compared with non-head-and-neck primaries (74% *vs.* 85%, *p* < 0.001) as were disease-specific survival rates (64% *vs.* 76%, *p* < 0.001). It is thought that worse disease control is a function of anatomic constraints limiting functional resections rather than a difference in biologic behavior and/or tumor histology. Osteosarcomas of the head and neck, compared with those presenting in long bones, arise in a somewhat older age group, have a higher risk for local recurrence, and a lower risk for distant metastasis [4].

We retrospectively analyzed Fox Chase Cancer Center’s experience with head and neck sarcomas during the past 10 years. We characterized a cohort of 37 patients with respect to their clinicopathologic features and course. We focused in particular on the results of treatment and identifying prognostic factors for DFS and OS in the era of contemporary multidisciplinary management. In general, whenever possible, patients with soft tissue sarcomas of the head/neck at our institution are treated with complete surgical resection. Recommendations for adjuvant chemotherapy and/or radiation are then made based on discussion at a multi-disciplinary conference which takes into account all clinical and pathologic features of a particular tumor, including histology. On the other hand, patients with osteosarcomas of the head/neck usually receive pre-operative chemotherapy followed by surgical resection. Again, decisions regarding adjuvant therapy are made based on final tumor size, histology and margin status.

## 2. Methods

Eligible patients were identified in the Center’s tumor registry. After receiving IRB approval, the medical records of all patients with head and neck sarcomas treated at Fox Chase Cancer Center between January 1999 and December 2009 were reviewed. The head and neck was defined as any site above the clavicles. Only patients whose diagnosis was confirmed by a pathology report were included in this study. Patients seen only for a second opinion, without treatment or follow-up at the Center, were excluded.

We recorded demographic and clinicopathologic characteristics including age, gender, presenting symptoms, tumor site, size, AJCC and MSKCC stage, histology, grade, margin status (if applicable) and sites of recurrence/metastases. Tumor grade at our institution is based on the French Federation Nationale des Centres de Lutte Contre le Cancer (FNCLCC) system. We also extracted data regarding treatment modality delivered (surgery, radiation and/or systemic therapy) at presentation and recurrence. Disease-free and overall survival were computed using the Kaplan-Meier method. DFS was measured starting on date of surgical resection and OS was measured starting on date of diagnostic biopsy. We were not able to accurately determine disease-specific survival (DSS) for our cohort since many patients were lost to follow-up after their initial treatment. Univariate analysis of clinicopathologic factors that could potentially affect survival were performed using the Cox proportional hazard model. The hazard ratio (HR) and 95% confidence intervals (CI) were reported.

## 3. Results for Soft Tissue Sarcoma Cohort

### 3.1. Patient and Tumor Characteristics

Between January 1999 and December 2009, 34 patients with head and neck sarcomas were seen. For the purposes of this study, four patients were excluded since they were only seen once for consultation with no follow-up information. Age at diagnosis ranged between 15 and 91 (median, 50 years). There were 20 male and 10 female patients. Four patients had a history of prior radiation exposure to the head and neck region for preexisting conditions and one patient had Gorlin’s syndrome (which is known to increase the risk of developing cancers).

Tumor site was grouped by the following anatomical regions: (a) scalp/face, (b) parotid/neck and (c) upper airway. Table 1 shows the patient distribution according to tumor site and histology. Most common were synovial cell sarcoma (6), rhabdomyosarcoma (5), angiosarcoma (4), liposarcoma (4) and leiomyosarcoma (3).

**Table 1:** Patient distribution according to tumor histology and site.

Histology	Scalp/Face	Parotid/Neck	Upper Airway	Total
Synovial cell sarcoma	3	3		6
Rhabdomyosarcoma	4		1	5
Angiosarcoma	4			4
Liposarcoma	1	3		4
Leiomyosarcoma	2		1	3
Spindle cell sarcoma	1	1	1	3
Alveolar soft part sarcoma			1	1
Carcinosarcoma	1			1
Epithelioid sarcoma	1			1
Fibrosarcoma	1			1
Histiocytic sarcoma	1			1
	19	7	4	30

Tumor size and grade were classified as per soft tissue sarcoma American Joint Committee on Cancer (AJCC) as well as Memorial Sloan Kettering Cancer Center (MSKCC) staging systems. Table 2 shows patient distribution according to these two parameters. 23 patients (77%) had high grade disease. The majority (28 of 30) presented with locoregional disease and only three had nodal involvement (all of these were rhabdomyosarcoma cases). Two patients had metastases at diagnosis (one case each of angiosarcoma and synovial cell).

**Table 2:** Patient distribution according to tumor size and grade.

Size	Grade				
	Low	Intermediate	High	Not Specified	Total
<5 cm	1	1	10	1	13
>5 cm	1	1	8	1	11
Unknown		1	5		6
Total	2	3	23	2	30

### 3.2. Treatment

Of 28 patients with localized disease, 24 underwent resection. In general, the surgeons performed an excision as wide as permitted by the nearby vital structures and functional concerns. Therefore, in this analysis, the surgical procedure is defined as gross total excision with final microscopic involved or R1 (7) and clear or R0 (17) margins. Seven patients underwent successful re-excision to achieve clear margins. Elective nodal dissection was not routinely performed in view of the low rate of regional node metastases in sarcomas. The four patients who did not have surgical excision included two angiosarcoma cases with locally advanced disease who received systemic chemotherapy and two rhabdomyosarcoma cases who were treated with combined modality chemotherapy and radiation.

Sixteen of 24 patients undergoing surgical resection also received radiation in an adjuvant (post-operative) fashion. Total tumor doses ranged from 5,000 cGy to 6,600 cGy; most patients received around 6,000 cGy over 30 fractions. IMRT (intensity-modulated radiation therapy) was utilized in all patients. Treatment volume was designed to cover the primary site plus a generous margin. The neck was not treated electively. Of the eight patients who did not receive radiation, five had tumors well below 5 cm in size with clear resection margins while two patients had received prior radiation which precluded them from additional therapy and one patient recurred within one month of resection. The median time interval between surgery and post-operative radiation therapy was 12 weeks.

Chemotherapy was administered to six patients, after completing post-operative radiation, on an individualized basis. These patients either had rhabdomyosarcoma (three cases), which is known to be sensitive to chemotherapy, or high-risk resected tumors (three cases) based on size and involved margins. Standard vincristine, adriamycin, cytoxan (VAC) chemotherapy was used for rhabdomyosarcoma cases and Ifosfamide-based therapy was used for other high-risk tumors.

### 3.3. Recurrence and Survival

Tumor recurrence was documented in sixteen patients: Seven locoregionally and at distant metastatic sites, five in the locoregional area alone and four in distant metastatic sites only. Sites of metastases included lung, liver and bones. Only two cases involved regional lymph node metastasis (angiosarcoma and rhabodomyosarcoma) which is not atypical for these sarcoma histologies. Over ninety percent of recurrences (15 of 16) occurred within the first 3 years. Median DFS for our cohort was 1.1 years (95% CI: 0.7–12.1) and median OS was 3.3 years (95% CI: 2.1–5.4).

In the recurrent and metastatic setting, 18 patients underwent salvage therapy in various combinations. Nine patients were submitted to repeat surgical resection, 10 received palliative radiation and 17 received standard or protocol-based systemic therapy. For patients with local or distant relapse, the 5-year survival with salvage treatment was dismal, at 24% in our series. More patients with local failure achieved long-term survival than those with distant spread.

### 3.4. Prognostic Factors

**Figure 1:**
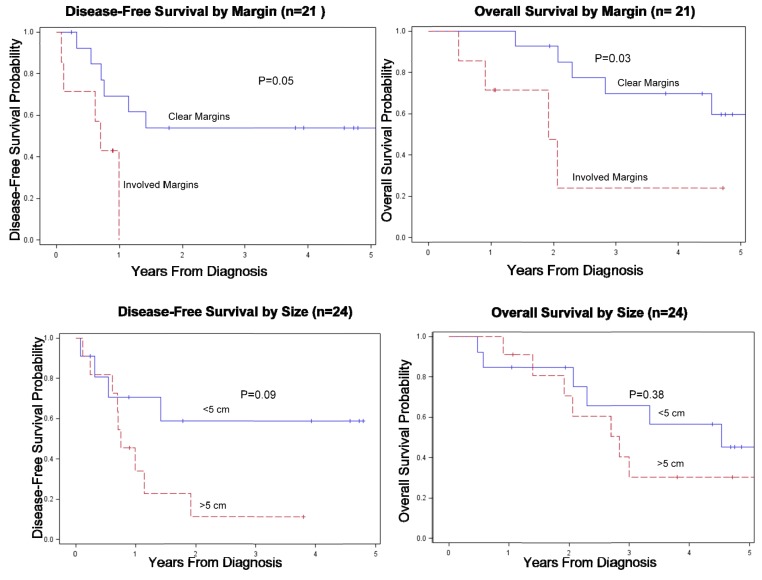
(**a**) DFS and OS according to margin status. (**b**) DFS and OS according to size in Soft Tissue Sarcoma Cases.

Univariate analysis using Cox progression hazard model was performed using margin status, size (<5 cm *vs.* >5 cm), age at diagnosis (<50 *vs.* >50), grade (low-intermediate *vs.* high) and multiple other recognized prognostic factors to identify predictors of DFS and OS. Patients with R1 resections had worse DFS (HR 3.74, 95% CI: 0.98–14.34) and OS (HR 4.4, 95% CI: 1.11–17.4). Tumors >5 cm were also associated with worse DFS (HR 2.79, 95% CI: 0.84–9.25). Age and grade did not correlate with outcome in our cohort. (See Table 3 and Figure 1).

**Table 3:** Univariate analysis of factors Associated WITH DFS and OS in soft tissue sarcomas.

Variable	Category	Number (n)	Disease-Free Survival		Overall Survival	
			HR (95% CI)	*p*-value	HR (95% CI)	*p*-value
Margin status	Clear	17	1.0	0.05	1.0	0.03
	Involved	7	3.74 (0.98, 14.34)		4.4 (1.11, 17.4)	
Size	<5 cm	13	1.0	0.09	1.0	0.38
	>5 cm	11	2.79 (0.84, 9.25)		1.63 (0.54, 4.9)	
Age at diagnosis	<50	12	1.0	0.77	1.0	0.3
	>50	18	0.85 (0.29, 2.5)		1.73 (0.64, 4.66)	
FNCLCC Grade	Lowintermed high	5	-	NS	-	NS
		23				
Gender	Male	20	1.0	0.16	1.0	0.18
	Female	10	0.46 (0.16, 1.35)		0.51 (0.19, 1.37)	
Site	Scalp/face	19	1.0	0.31	1.0	0.97
	Parotid/neck	7	0.77 (0.20, 2.93)		0.61 (0.17, 2.18)	
	Upper Airway	4	2.40 (0.69, 8.32)		1.32 (0.36, 4.78)	
Extension	Superficial	4	1.0	0.1	1.0	0.97
	Deep	26	1.49 (0.19, 11.37)		0.98 (0.28, 3.45)	
Radiation	No	10	1.0	0.14	1.0	0.07
	Primary	2	1.78 (0.32, 9.78)		0.55 (0.31, 0.97)	
	Adjuvant	16	0.45 (0.14, 1.43)		0.34 (0.11, 1.02)	
Chemotherapy	No	18	1.0	0.6	1.0	0.09
	Primary	4	3.35 (0.67, 16.71)		0.48 (0.21, 1.1)	
	Adjuvant	6	1.24 (0.33, 4.64)		1.29 (0.4, 4.14)	
AJCC	I and II	10	1.0	0.021	1.0	0.27
	III	12	2.24 (1.13, 4.44)		2.59 (0.74, 8.98)	
	IV	8	6.29 (1.2, 33.06)		2.14 (0.57, 8.02)	
MSKCC	0 and I	5	1.0	0.11	1.0	0.14
	II	7	2.2 (0.84, 5.8)		1.79 (0.82, 3.88)	
	III and IV	18	2.25 (0.29, 17.76)		1.61 (0.45, 5.76)	
N1 or M1	No	22	1.0	0.11	1.0	0.67
	Yes	8	2.39 (0.81, 7.02)		1.24 (0.47, 3.3)	
TNM	T1aN0M0-T1bN1M1	12	1.0	0.18	1.0	0.07
	T2aN0M0-T2bN1M1	18	2.2 (0.7, 6.96)		2.9 (0.93, 9.08)	

## 4. Results for Osteogenic Sarcoma Cohort

### 4.1. Patient and Tumor Characteristics

From 1999–2009, seven patients were treated for an osteosarcoma of the head and neck at Fox Chase Cancer Center. All were localized at diagnosis (AJCC Stage IA: four patients, IIA: two patients, IIB: one patient). Median age at diagnosis was much younger than that of soft tissue sarcoma (27.1 years, range 18.6–46.8) and all but one tumor was located in the mandible. One case was a chondroblastic osteosarcoma, while all others were osteosarcoma NOS.

### 4.2. Treatment

A total of 71% (5/7) patients received pre-operative systemic therapy generally consisting of cisplatin and an anthracycline +/− high dose methotrexate. Two patients were not given pre-operative chemotherapy based on low grade and small size of their tumors. Although not customarily described in the pathologic report, it seems only one tumor (Stage IIA of the mandible) had an excellent response to induction chemotherapy (95% necrosis). Forty-three percent (three of seven) of patients received adjuvant systemic therapy, generally with similar agents. Of the four patients who did not receive adjuvant chemotherapy, one had a low grade tumor, one patient refused and reasons for not administering adjuvant chemotherapy for the remaining two cases are unclear.

All patients with mandibular osteosarcomas underwent composite resection of the jaw and necessary soft tissue with reconstruction as a definitive surgical procedure. There were no elective neck dissections, but neck vessels were exposed in the course of free tissue transfer. One patient with a maxillary primary tumor was submitted to infrastructure maxillectomy, with subsequent fabrication of a custom obturator. Margins were clear in three patients, close (≤1 mm) in two patients and involved by cancer in two patients. All reconstructions were successful, although two patients had complications requiring additional operative procedures.

Involved and/or close margins were considered indications for adjuvant radiotherapy. Weekly low dose cisplatin (30 mg/m^2^) was administered with radiation. Both patients with involved margins and one of two patients with a close margin received adjuvant chemoradiation (median dose 6,600 cGy, range 6,000–6,600 cGy). There were no ≥grade 3 radiation complications.

### 4.3. Recurrence and Survival

The median follow-up was 3.1 years (range 1.0–5.1 years). There were two recurrences, both of which were local recurrences within a year of completion of therapy. One recurrence was in a patient with high grade tumor and an involved margin who received adjuvant radiation and systemic chemotherapy. The other failure was of a low grade lesion with a clear margin who had received standard cisplatin and anthracycline chemotherapy (three cycles prior to surgery and two cycles after; the planned 6th cycle was not delivered secondary to disease recurrence). Both patients died of disease at 1.6 and 0.7 years from completion of therapy respectively. Crude locoregional control was therefore 71%, and not affected by margin status (involved/close margin locoregional control: 75%; clear margin locoregional control: 67%).

## 5. Discussion

There have been few reports addressing the behavior and treatment of head and neck sarcomas in adults. This report represents an effort to describe outcomes and identify important prognostic factors among a contemporary cohort.

The 5-year disease-free and overall survival for our cohort of soft tissue sarcoma patients was 35% and 46%, slightly lower than suggested in the literature [5,6,7,8,9,10,11,12], perhaps because a majority (23 of 30) of our patients had high grade tumors. Another difference we noted was a higher percentage of synovial cell sarcomas compared with other series. This is unusual and may be due to our purely adult population. Synovial cell histology did not predict for patient outcome but analysis is limited due to the small number of cases. On univariate analysis, margin status and size were associated with survival, consistent with previous findings [6,13,14,15,16,17,18]. These factors seem related because it is more difficult to derive clear surgical margins with larger tumors—but our numbers do not permit multivariate analysis. This finding emphasizes that every thorough effort should be made to resect these tumors completely in the initial resection, including the use of modern reconstruction techniques. This is best accomplished at high-volume surgical centers with accomplished head and neck and reconstructive surgeons as well as experienced members of adjunctive services such as speech-language pathologists. Returning to high quality of life after *en bloc* resection about the head and neck region is demanding because of functional and cosmetic sequelae of the necessary resection. However, the extent and adequacy of excision may inform survival and incidence of local recurrence.

Unlike other series [5,12,19,20,21], we did not find age or grade to be prognostic factors for disease control. This may be attributed to our sample size and larger number of patients with high grade tumors. Higher-grade sarcomas are more aggressive than low grade disease, recurring locally as well as metastasizing, thus leading to poorer outcomes.

Surgery remains the mainstay of treatment for head and neck sarcomas. Noteworthy exceptions to this principle include most rhabdomyosarcomas and Ewing’s sarcomas. Radiation therapy is indicated after resection of all high grade sarcomas, large tumors, and when margins of resection are close or microscopically involved. There was no difference in local control or overall survival between patients in our series who were given postoperative radiotherapy and those who were not. This suggests that radiation is effective since the group that received adjuvant radiation had proportionally more aggressive tumors. Systemic chemotherapy is recommended for those tumors with a significant risk of distant metastases.

High local failure rates in the head and neck have historically been associated with poorer treatment outcomes in this group. Median DFS and OS in our series are worse compared to those achieved with other sub-sites [3,22,23,24], despite the finding that sarcomas arising in the head and neck have a lower probability of distant spread. This is commonly attributed to earlier diagnosis with a higher proportion of small tumors.

At disease relapse, repeat surgery and/or radiation should be considered to maximize control [25,26]. In addition, enrollment of these sarcoma patients to clinical trials investigating novel systemic therapies remains a priority.

Although limited by a small number of patients in our study, head and neck osteosarcoma appears to be a different disease than soft tissue sarcoma. It affects younger patients than does soft tissue sarcoma, is not as frequently of high grade, and appears to have a better prognosis. Most patients had lesions of the mandible, which along with the maxilla, is the most common site of primary tumors [4,27]. Due to small numbers, we were unable to determine prognostic factors (size, margin status or grade) predictive of recurrence. A large retrospective series from MD Anderson, however, indicated that high grade tumors, radiation induced tumors, and involved margins were associated with a poorer disease free survival and overall survival at five years [27]. Patients in that series with involved or uncertain margins who received chemotherapy had improved outcomes compared to those who were treated with surgery alone. They demonstrated no benefit from radiation alone, however only 23% of the patients were treated with radiation in the postoperative setting. Our institutional experience supports a policy of surgical extirpation with reconstruction, systemic therapy, and risk adapted use of external beam radiotherapy. None of our patients developed distant metastases, but there are reports with up to 21% of patients with distant progression (primarily to lung, bone, and brain) [27,28].

## 6. Conclusions

Patients with head and neck sarcomas should undergo wide excision with emphasis on removal of gross disease and attaining clear surgical margins. In most patients, except those with small, low grade lesions, postoperative radiation therapy should be added to maximize local control. Head and neck sarcomas are rare tumors that can present management difficulties. These tumors are best managed in a multi-disciplinary setting.

## References

[1] Potter B.O., Sturgis E.M. (2003). Sarcomas of the head and neck. Surg. Oncol. Clin. N. Am..

[2] Hoffman H.T., Robinson R.A., Spiess J.L., Buatti J. (2004). Update in management of head and neck sarcoma. Curr. Opin. Oncol..

[3] Zagars G.K., Ballo M.T., Pisters P.W., Pollock R.E., Patel S.R., Benjamin R.S., Evans H.L. (2003). Prognostic factors for patients with localized soft-tissue sarcoma treated with conservation surgery and radiation therapy: An analysis of 1,225 patients. Cancer.

[4] Kassir R.R., Rassekh C.H., Kinsella J.B., Segas J., Carrau R.L., Hokanson J.A. (1997). Osteosarcoma of the head and neck: Meta-analysis of nonrandomized studies. Laryngoscope.

[5] Willers H., Hug E.B., Spiro I.J., Efird J.T., Rosenberg A.E., Wang C.C. (1995). Adult soft tissue sarcomas of the head and neck treated by radiation and surgery or radiation alone: Patterns of failure and prognostic factors. Int. J. Radiat. Oncol. Biol. Phys..

[6] Kraus D.H., Dubner S., Harrison L.B., Strong E.W., Hajdu S.I., Kher U., Begg C., Brennan M.F. (1994). Prognostic factors for recurrence and survival in head and neck soft tissue sarcomas. Cancer.

[7] Dijkstra M.D., Balm A.J., Coevorden F.V., Gregor R.T., Hart A.A., Hilgers F.J., Keus R.B., Loftus B.M. (1996). Survival of adult patients with head and neck soft tissue sarcomas. Clin. Otolaryngol..

[8] Barker J.L., Paulino A.C., Feeney S., McCulloch T., Hoffman H. (2003). Locoregional treatment for adult soft tissue sarcomas of the head and neck: An institutional review. Cancer J..

[9] Le Vay J., O’Sullivan B., Catton C., Cummings B., Fornasier V., Gullane P., Simm J. (1994). An assessment of prognostic factors in soft tissue sarcoma of the head and neck. Arch. Otolaryngol. Head Neck Surg..

[10] Le Q.T., Fu K.K., Kroll S., Fitts L., Massullo V., Ferrell L., Kaplan M.J., Phillips T.L. (1997). Prognostic factors in adult soft-tissue sarcomas of the head and neck. Int. J. Radiat. Oncol. Biol. Phys..

[11] Chen S.A., Morris C.G., Amdur R.J., Werning J.W., Villaret D.B., Mendenhall W.M. (2005). Adult head and neck soft tissue sarcomas. Am. J. Clin. Oncol..

[12] Penel N., Mallet Y.M., Robin C., Fournier C., Grosjean J., Ceugnart L., Clisant S., Lefebvre J.L. (2008). Prognostic factors for adult sarcomas of head and neck. Int. J. Oral Maxillofac. Surg..

[13] Farhood A.I., Hajdu S.I., Shiu M.H., Strong E.W. (1990). Soft tissue sarcomas of the head and neck in adults. Am. J. Surg..

[14] Tran L.M., Mark R., Meier R., Calcaterra T.C., Parker R.G. (1992). Sarcomas of the head and neck: Prognostic factors and treatment strategies. Cancer.

[15] Weber R.S., Benjamin R.S., Peters L.J., Ro J.Y., Achon O., Goepfert H. (1986). Soft tissue sarcomas of the head and neck in adolescence and adults. Am. J. Surg..

[16] De Bree R., van der Valk P., Kuik D.J., van Diest P.J., Doornaert P., Buter J., Eerenstein S.E., Langendijk J.A., van der Waal I., Leemans C.R. (2006). Prognostic factors in adult soft-tissue sarcomas of the head and neck: A single-centre experience. Oral Oncol..

[17] Van Damme J.P., Schmitz S., Machiels J.P., Galent C., Gregoire V., Lengele B., Hamoir M. (2010). Prognostic factors and assessment of staging systems for head and neck soft tissue sarcomas in adults. EJSO.

[18] Gonzalez-Gonzales R., Bologna-Molina N., Molina-Frechero H.R., Dominguez-Malagon H.R. (2012). Prognostic factors and treatment strategies for adult head and neck soft tissue sarcoma. Int. J. Oral. Maxillofac. Surg..

[19] Greager J.A., Patel M.K., Briele H.A., Walker M.J., Das Gupta T.K. (1985). Soft tissue sarcomas of the head and neck. Cancer.

[20] Bentz B.G., Singh B., Woodruff J., Brennan M., Shah J.P., Kraus D. (2004). Head and neck soft tissue sarcomas: A multivariate analysis of outcomes. Ann. Surg. Oncol..

[21] Huber G.F., Matthews T.W., Dort J.C. (2006). Soft tissue sarcomas of the head and neck: A retrospective analysis of the Alberta experience 1974 to 1999. Laryngoscope.

[22] Robinson M., Barr L., Fisher C., Fryatt I., Stotter A., Harmer C., Wiltshaw E., Westbury G. (1990). Treatment of extremity soft tissue sarcomas with surgery and radiotherapy. Radiother. Oncol..

[23] Sadoski C., Suit H.D., Rosenberg A., Mankin H., Efird J. (1993). Preoperative radiation, surgical margins and local control of extremity sarcomas of soft tissues. J. Surg. Oncol..

[24] Suit H.D., Mankin H.J., Wood W.C., Proppe K.H. (1985). Preoperative, intraoperative and postoperative radiation in the treatment of primary soft tissue sarcoma. Cancer.

[25] Mendenhall W.M., Mendenhall C.M., Werning J.W., Riggs C.E., Mendenhall N.P. (2005). Adult head and neck soft tissue sarcomas. Head Neck.

[26] Dudhat S.B., Mistry R.C., Varughese T., Fakih A.R., Chinoy R.F. (2000). Prognostic factors in head and neck soft tissue sarcomas. Cancer.

[27] Guadagnolo B.A., Zagars G.K., Raymond A.K., Benjamin R.S., Sturgis E.M. (2009). Osteosarcoma of the jaw/craniofacial region outcomes after multimodality treatment. Cancer.

[28] Chennupati S.K., Norris R., Dunham B., Kazahaya K. (2008). Osteosarcoma of the skull base: Case report and review of literature. Int. J. Ped. Otorhinolaryngol..

